# Templated chemistry for bioorganic synthesis and chemical biology

**DOI:** 10.1002/psc.3198

**Published:** 2019-07-15

**Authors:** Oliver Seitz

**Affiliations:** ^1^ Department of Chemistry Humboldt University Berlin Berlin Germany

**Keywords:** Oligonucleotides, carbohydrates, lectin, multivalency, bioconjugation, peptide ligation, protein labeling, templated synthesis

## Abstract

In light of the 2018 Max Bergmann Medal, this review discusses advancements on chemical biology–driven templated chemistry developed in the author's laboratories. The focused review introduces the template categories applied to orient functional units such as functional groups, chromophores, biomolecules, or ligands in space. Unimolecular templates applied in protein synthesis facilitate fragment coupling of unprotected peptides. Templating via bimolecular assemblies provides control over proximity relationships between functional units of two molecules. As an instructive example, the coiled coil peptide–templated labelling of receptor proteins on live cells will be shown. Termolecular assemblies provide the opportunity to put the proximity of functional units on two (bio)molecules under the control of a third party molecule. This allows the design of conditional bimolecular reactions. A notable example is DNA/RNA–triggered peptide synthesis. The last section shows how termolecular and multimolecular assemblies can be used to better characterize and understand multivalent protein‐ligand interactions.

## 1 INTRODUCTION

The molecules used in chemical biology studies face a specificity challenge: The envisioned probe/drug ought to recognize and exert action only upon a selected target that will be a minority compound in the vast space of cell's biomolecules. This problem becomes most pronounced when the probe/drug must not only recognize or bind to its biomolecule target but must also initiate a chemical reaction. Such reactions are used for labelling of biomolecules with reporter groups or affinity tags to visualize their localization and understand their functions in the native environment.^1^, ^2^, ^3^, ^4^, ^5^ However, the functional groups offered by biomolecules do not lend themselves to target specific covalent labelling unless a particular microenvironment such as an enzyme's active site facilitates a regioselective reaction that is the basis of activity‐based profiling.^6^, ^7^ Most biomolecules, however, lack such reactivity enhancing microenvironments. In this case, artificial functional groups can be introduced by metabolic engineering or expanded genetic code technologies.^8^, ^9^, ^10^, ^11^, ^12^ Specificity problems also complicate the targeting of specific binding sites that are common to a family of related but functionally distinct biomolecules. This situation frequently emerges for protein‐based receptor targets that rely on multivalent interactions with low affinity ligands such as carbohydrates or peptide motive repeats.^13^, ^14^, ^15^

One solution to this lack of target specificity is to utilize templates, designer scaffolds that orient functional units in space. The spatial arrangement and not the functional unit per se is crucial for the template action. For example, a template can organize ligands to match the orientation of binding sites of a receptor. Templating can bring functional groups into proximity to direct or enable reactions that would otherwise not occur or at least not in a target‐specific fashion. Prominent examples can be found in nucleic acid directed chemistry where the sequence‐specific interactions between complementary oligonucleotides increase the effective molarity of functional groups and enable chemical reactions under dilute conditions where nontemplated biomolecular reactions cannot occur.^16^, ^17^, ^18^, ^19^

Focusing on own work, this article intends to categorize and showcase different templating approaches used in bioorganic synthesis and chemical biology.

## 2 TEMPLATES

**Figure 1:**
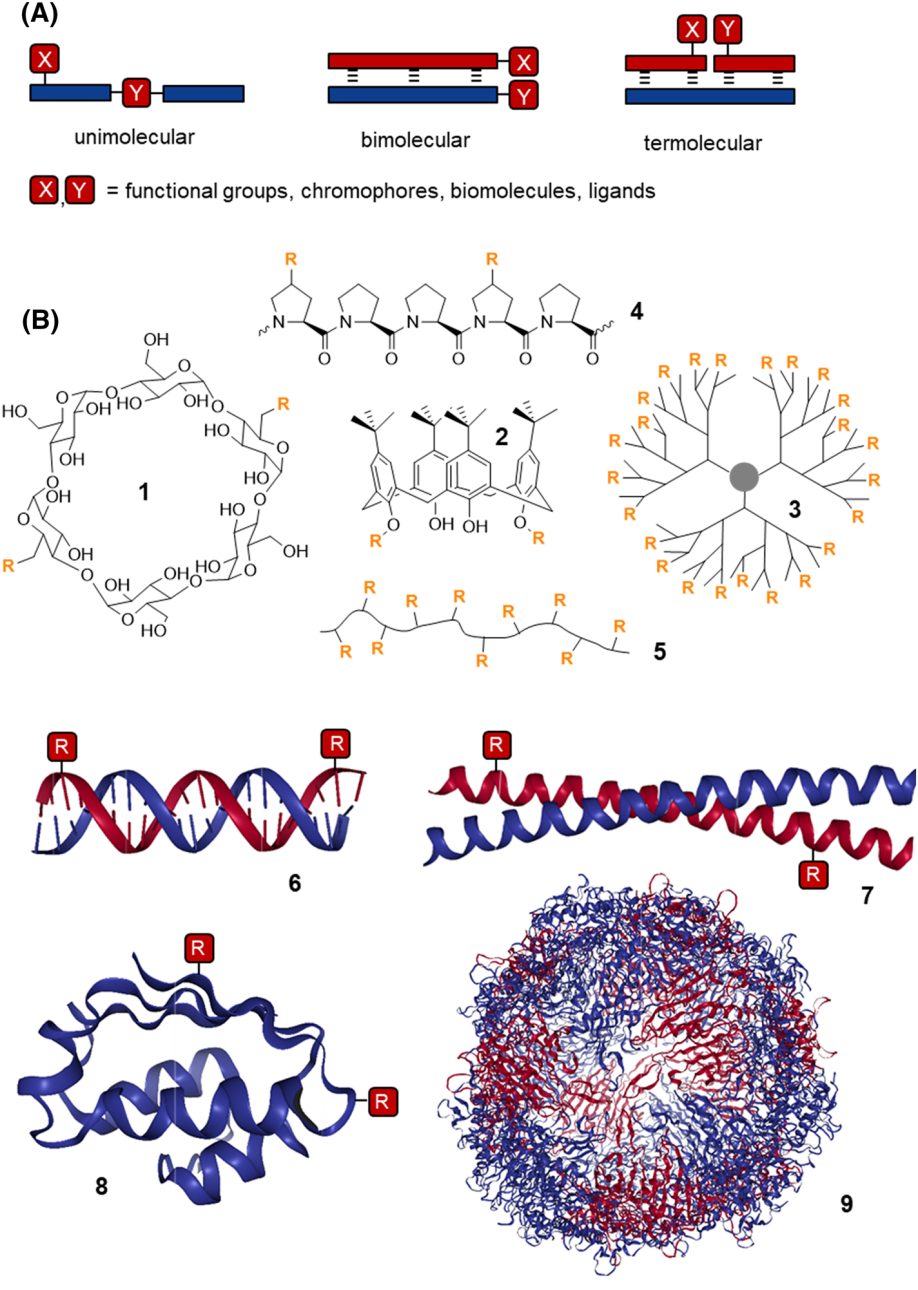
A, Templating strategies to arrange the functional units X and Y in defined orientation and distance. B, Representative examples of template molecules used for presentation of groups R (PDB IDs: **6**, 1LAI; **7**, 2XU6; **8**, 1BTA; **9**, 2VTU)

One way to categorize templating strategies used in bioorganic chemistry and chemical biology refers to the molecularity of the template complex. For simplicity, only two functional units X and Y are shown in Figure 1A. These units may represent (i) functional groups that engage in a templated reaction, (ii) dyes or other reporter groups that engage in distance‐dependent interactions, or (iii) ligands or biomolecules.

A unimolecular template connects the functional units within a single molecular entity that may serve as a stand‐alone scaffold or may be conjugated with another molecular unit. Typical examples (Figure 1B) for stand‐alone templates are small molecule scaffolds such as cyclodextrins (**1**),^20^ calixarenes (**2**),^21^ and many more as well as larger molecules such as dendrimers (**3**),^22^, ^23^, ^24^ peptides (including oligoproline **4**),^25^, ^26^, ^27^, ^28^, ^29^ or even entire proteins (**8**)^30^ or polymers (**5**).^31^, ^32^ In another application scenario, conjugated unimolecular templates are frequently used as reaction scaffolds to facilitate chemical reactions such as ligation auxiliaries in protein synthesis via native chemical ligation (NCL) (*vide infra*).^33^, ^34^, ^35^

Templating via bimolecular assembly relies on the mutual interactions between two components. Each component presents a functional unit X or Y. The recognition event defines the proximity relationship between the two components. Instructive examples for bimolecular templates include dual pharmacophore DNA‐encoded libraries,^36^ proximity‐triggered methods for protein labelling,^37^, ^38^ and chemical noncovalent protein dimerization.^38^, ^39^, ^40^

Nucleic acid– and protein‐based molecules can be designed to engage in termolecular or even multimolecular template complexes. If only two components carry functional units, the third component typically serves as a landing hub that orchestrates the proximity between X and Y. The larger the landing hub component the easier it is to extend this approach to multimolecular complexes. For example, long DNA‐type strands have been used to construct multivalent ligand assemblies by recruitment of multiple ligand‐modified oligonucleotide strands.^41^, ^42^ In some applications, each component interacts with at least two other components. This principle has been used for the design of multivalent carriers on the basis of multimolecular protein assemblies such as viral capsids (**9**).^43^, ^44^

The use of the term “template” implies that the molecular architecture provides a certain degree of structural integrity required for orienting the functional units in space. This is comparably easy to achieve with small molecules such as benzene derivatives, monosaccharides, calixarenes, cyclodextrins, and other macrocycles, which allow confinement of functional units within <20 Å distance. Dendrimeric structures (**3** in Figure 1B) have frequently been used for clustering.^45^, ^46^, ^47^, ^48^ A precise presentation of functional units over larger distances is more difficult to achieve. This is the domain of nucleic acid– and protein‐based scaffolds, which form accurately defined tertiary structures. For example, DNA duplex (**6**), triplex, and quadruplex structures are rigid and can be fashioned to have high persistence length over >150 Å distances.^42^ DNA origami allows the construction of nanosized templates with sequence‐programmed three‐dimensional shape.^49^, ^50^ Peptide‐based coiled coils (**7**)^51^ and oligoproline scaffolds^29^ fold into helical structures that enable the spatially defined arrangement of functional units along a two‐dimensional track. Although de novo design of protein 3D objects is rapidly improving,^52^ folded protein scaffolds (**8**) from natural sources and viral capsids (**9**) are, perhaps, currently preferable for a three‐dimensional presentation of functional units.

## 3 UNIMOLECULAR TEMPLATES AS LIGATION SCAFFOLDS IN PROTEIN SYNTHESIS

**Figure 2:**
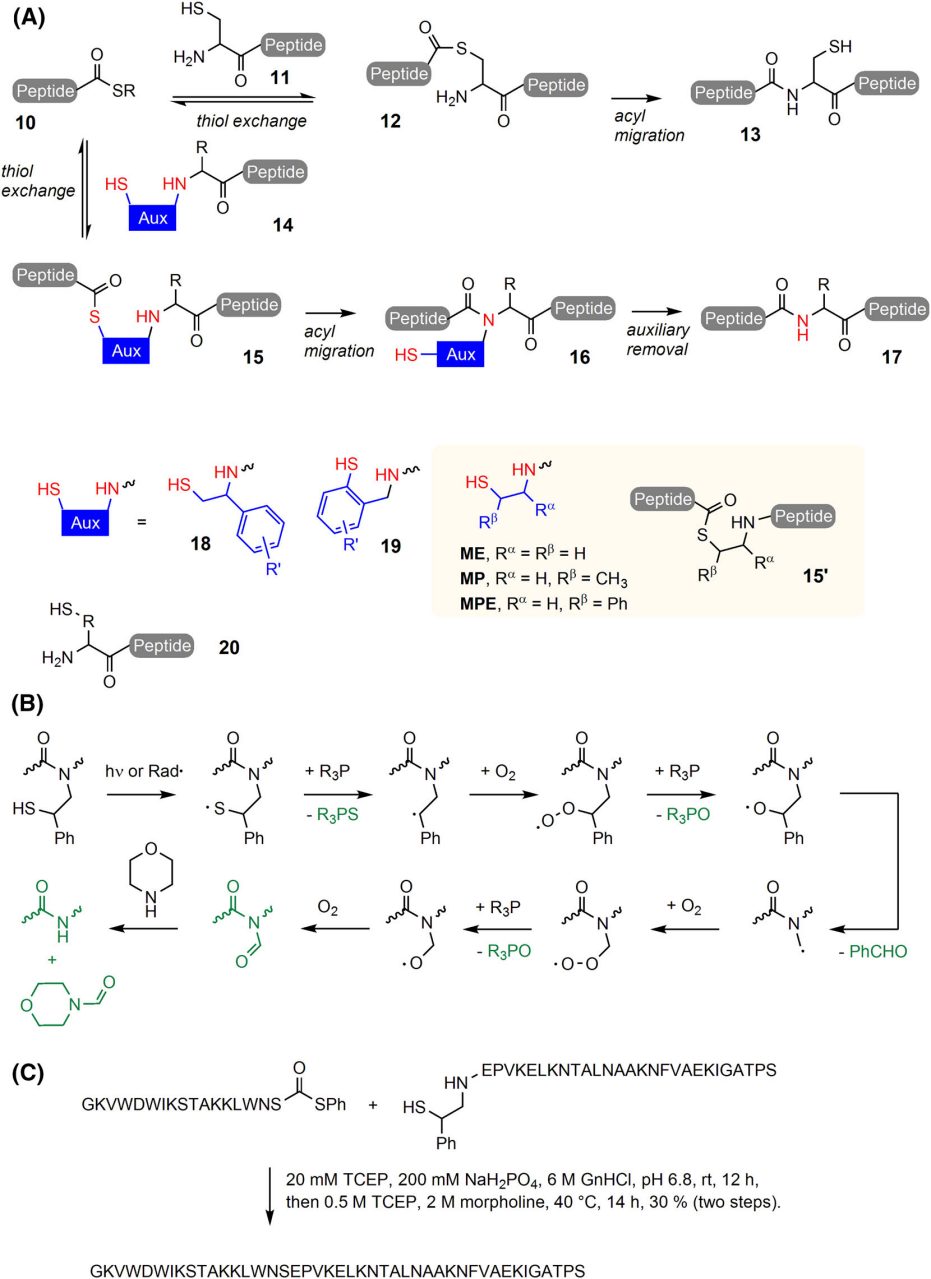
A, Native chemical ligation (**10** + **11**) and auxiliary mediated native chemical ligation (**10** + **14**). B, Proposed radical‐induced oxidative fragmentation of the MPE auxiliary. Products highlighted in green were detected by high‐performance liquid chromatography–mass spectrometry (HPLC‐MS) and nuclear magnetic resonance (NMR). C, One‐pot ligation auxiliary removal in the synthesis of opistoporin‐2 (GnHCl, guanidinium hydrochloride; TCEP, triscarboxyethylphosphine)

Modern chemical synthesis of proteins depends upon ligations of unprotected peptide fragments, which are easier to handle than protected segments because of their higher solubility. Until the development of NCL^53^ chemistry, the selective chemical coupling of two unprotected peptides was a major challenge. NCL reactions involve a C‐terminal peptide thioester **10** and a cysteinyl residue **11** at the N‐terminus of the C‐terminal segment (Figure 2A). The cysteine residue adopts the function of an intrinsic template. Owing to a chemoselective thiol exchange reaction, the side chain mercapto group captures the acyl component in the form of an intermediary thioester **12**, which subsequently rearranges via an intramolecular S‐N acyl shift to the ligation product **13**. For proteins that lack cysteine, the side chain may be emulated by means of an auxiliary (**14**)^54^ that is appended to the peptide. Mercapto groups for acyl capture have also been anchored via ester bonds to glutamate^55^ and glycan residues.^56^ The most versatile chemistry has been introduced by Offer et al^57^ and Botti et al^34^ who connected benzyl‐type scaffolds **18** and **19** to the N‐terminal amine. The approach is attractive because the ligation auxiliary is introduced in the last step of solid‐phase peptide synthesis via reductive alkylation. Preformed amino acid building blocks are not required. Electron‐donating substituents at the aryl part facilitate the removal of the ligation template upon acid treatment. However, ligation at the benzyl‐type templates proceeds with rather low rates, which restricts their application to glycine‐containing ligation junctions. This probably is the reason why an alternative approach, the ligation‐desulfurization method,^58^, ^59^, ^60^, ^61^ gained popularity. The mercapto group is attached directly to the side chain of the N‐terminal amino acid in **20** and is removed after ligation by a radical desulfurization^62^ reaction. This method allowed faster ligation reactions. A drawback is the additional workload required for the preparation of the thiolated amino acid building blocks.

Motivated by the prospect of extending NCL without the need for new amino acid building blocks, we rethought the ligation auxiliary approach. A suitable ligation template should provide high reactivity for both the thiol exchange and the S → N acyl migratory steps and must lend itself to facile removal under conditions that leave the ligation product unharmed. We systematically analyzed the architecture of the ligation template and found that the substituents at the α‐ and β‐positions in **15′** play key roles.^35^, ^63^ A comparison of the three mercaptoethyl templates ME, MP, and MPE showed, perhaps surprisingly, that the additional β‐substituents in MP and MPE accelerated the ligation. In unpublished work, however, we observed that geminal bissubstitution at the β‐position ought to be avoided. After an evaluation of nine different structures, we noticed that ligations were fastest for templates that lacked an α‐substituent. Apparently, steric crowding around the amino group in the tetrahedral intermediate **15'** is detrimental. This finding was concerning because in traditional ligation auxiliaries, α‐substitution was a requirement for cleavability (phenyl substitution in benzyl‐type template). However, we discovered a potentially general approach for the removal of N‐amide–linked mercaptoethyl groups. Nuclear magnetic resonance (NMR) analysis of a C^13^‐labelled ligation template suggested that treatment with phosphine in aqueous morpholine at pH 8.5 triggers a radical‐induced oxidative fragmentation (Figure 2B).^35^ The investigation led us to the 2‐mercapto‐2‐phenethyl (MPE) template, which is the first ligation auxiliary that enables NCL at ligation sites beyond glycine. We have proven the usefulness of MPE in the chemical total synthesis of the 48 amino acid residue peptide opistoporin‐2 (Figure 2C)^63^ and a 126 amino acid long mucin‐1 protein.^64^

## 4 BIMOLECULAR ASSEMBLIES FOR TEMPLATED LABELLING OF PROTEINS ON THE SURFACE OF LIVE CELLS

The labelling of proteins in living cells or tissues is a key enabling method for the cell biological sciences. Fluorescent reporter groups allow the visualization of protein localization and trafficking by fluorescence microscopic methods. Combinations of fluorescence labels enable studies of protein interactions, and time‐resolved labelling/photobleaching provides information about dynamic properties.

Genetic fusions with autofluorescent proteins or self‐labelling enzymes make protein fluorescent labelling readily available for nonchemists.^65^, ^66^ While useful in many cases, the large size (18‐33 kDa) of these reporters can impair the functional properties of the protein of interest. This limitation motivated intense research efforts geared towards the development of labelling methods that proceed with smaller tags.^37^

**Figure 3:**
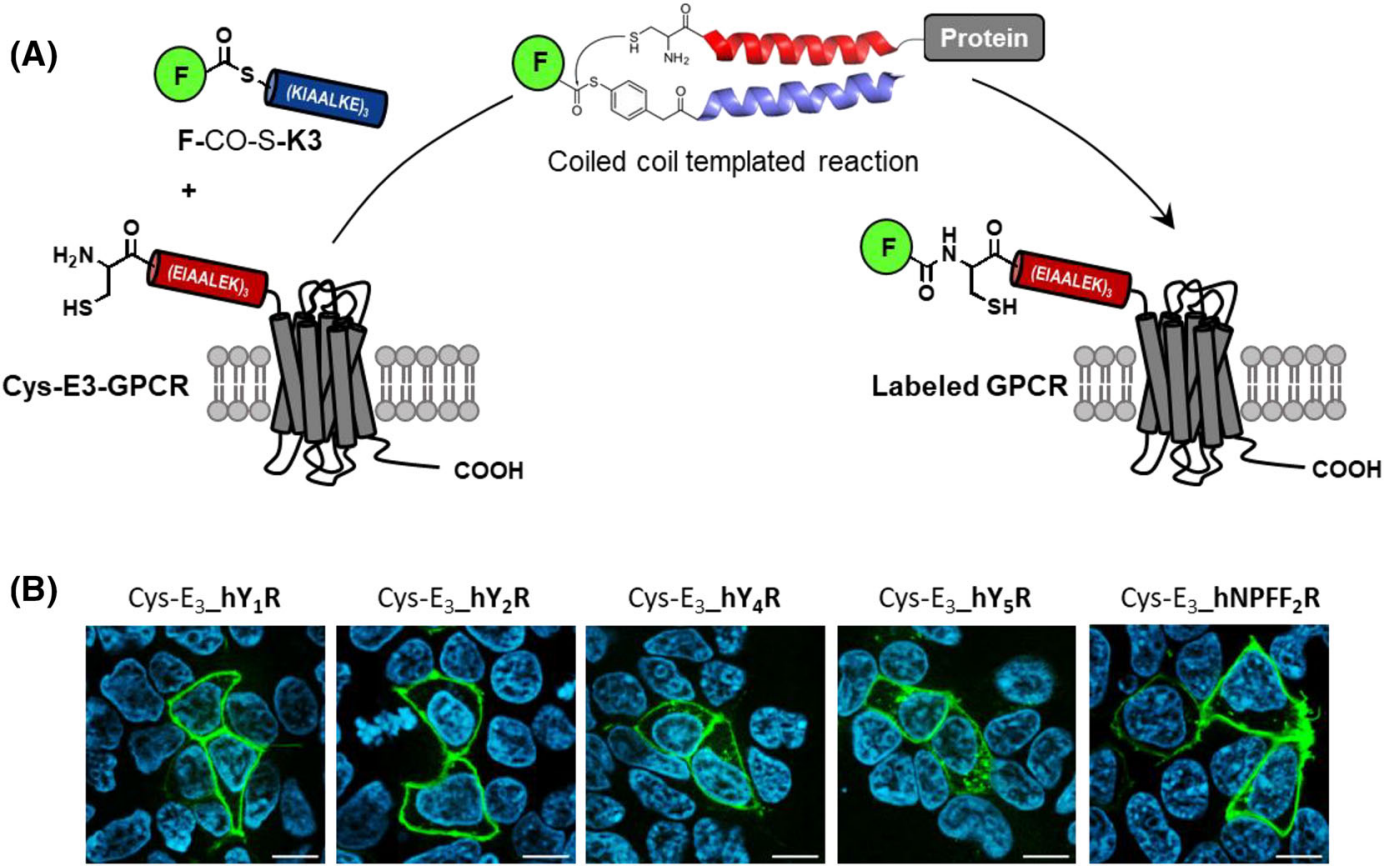
A, Formation of the E3‐K3 coiled coil allows the rapid and selective labelling of a Cys‐E3 tagged GPCR by thioester‐linked fluorophore‐K3 peptide conjugates. B, Live cell fluorescence microscopy images after 5‐min incubation of transiently transfected HEK293 cells (Cys‐E3_hY_1_R, Cys‐E3_hY_2_R, Cys‐E3_hY_4_R, Cys‐E3_hY_5_R, and Cys‐E3_hNPFF_2_R) with 100 nM ATTO488‐K3 conjugate (reproduced from Reinhardt et al^68^ copyright 2015 American Chemical Society)

We have introduced a peptide‐templated labelling reaction that relies on the formation of a peptide coiled coil complex.^67^, ^68^ The coiled coil motif involves two (or more) α‐helices that wrap around each other. Our work was inspired by contributions from Matsuzaki, who applied the artificial heterodimeric coiled coil peptides E3 and K3, initially developed by Litowski and Hodges,^69^ for the noncovalent labelling of cell surface proteins.^70^ We equipped the 21 aa long E3 peptide with a N‐terminal cysteine residue. This peptide was used as the genetically encoded tag (see Cys‐E3‐GPCR, Figure 3A). A modified K3 peptide served as the labelling agent. For this purpose, a fluorophore is connected via a thioester linkage with the N‐terminus (see **F‐**CO‐S‐**K3**). The formation of the E3‐K3 parallel coiled coil brings the thioester unit into close proximity with the cysteinyl residue. This arrangement templates an acyl transfer reaction, which proceeds in analogy to an NCL reaction. The end‐of‐helix arrangement of the functional groups results in a very high effective molarity. Furthermore, arylmercapto‐linked thioesters are known to react rapidly in NCL reactions.^71^ Therefore, formation of the coiled coil triggers an almost instantaneous labelling reaction. The E3‐K3 coiled coil has a stability in the nanomolar range (*K*_d_ = 70 nM). As a result, the E3‐K3–templated labelling reaction proceeds rapidly at 100 nM concentration of reactants. At concentrations this low, nontemplated acylation reactions are negligible. The labelling reaction occurs with high target specificity, proceeds within seconds to minutes, and offers a free choice of the transferred reporter group.

We have used the coiled coil–templated acyl transfer for the labelling of cell surface proteins expressed in HEK293 cells and Chinese hamster ovary (CHO) cells. Labelling of G‐protein coupled receptors (GPCRs) such as human neuropeptide Y receptors 1, 2, 4, 5, human dopamine receptor and human neuropeptide FF receptors 1 and 2 succeeded within 2 to 5 minutes by using 100nM labelling agent only (Figure 3B). We demonstrated labelling with AF350, ATTO488, TAMRA, and biotin. The method facilitates the analysis of GPCR trafficking. In one example, we followed the intracellular transport of internalized human neuropeptide Y2 receptors (hY_2_R).^72^ Unstimulated HEK cells expressing the tagged hY_2_R were incubated with a TAMRA‐labelling agent. Treatment with the neuropeptide Y2 triggered internalization. At the concentration applied internalization was not quantitative. The remaining receptors were labelled with ATTO488. After a second stimulation with neuropeptide Y2, the spatial distribution of vesicles containing TAMRA and/or ATTO488 labels was followed by confocal fluorescence microscopy. The two‐color pulse‐chase experiment revealed that rather than traveling separately, vesicles from separately internalized hY_2_R begin to fuse already after 10 to 12 minutes. Fusion was complete after 30 minutes, and the receptors were pooled in Rab‐4–positive vesicles for fast recycling.

In ongoing work, we explore the reversibility of coiled coil formation^73^ and develop methods for tagging proteins with peptide nucleic acid (PNA) strands, which is expected to provide a universal platform for labelling and manipulation of cell surface proteins.

## 5 TERMOLECULAR ASSEMBLIES FOR NUCLEIC ACID–PROGRAMMED PEPTIDE SYNTHESIS

It is a fascinating vision to allow chemical reactions to only occur in or on a selected subset of cells. Such a reaction could lead to the formation of cell toxic molecules that would be formed only in diseased cells, eg, cancer cells. To achieve such cell‐type specificity, the chemical reaction must be conditional and require a specific trigger that occurs only in the selected cell type. Given that a cell's RNA expression profile encodes its phenotype and considering the breadth of DNA‐encoded chemistries available today, nucleic acid templates seem like an appropriate trigger. Taylor was among the first to describe a reaction system that could, in principle, allow a nucleic acid triggered drug release.^74^ The reaction was based on the hydrolysis of a nitrophenyl ester. Today, many more nucleic acid–templated reactions are available including, among others, nucleophilic displacements,^75^, ^76^ tetrazine‐triggered reactions,^77^, ^78^ Staudinger reductions,^79^, ^80^, ^81^ bisarsenic thioester formation,^82^ olefination reactions,^83^, ^84^, ^85^ and photoinduced oxidative and reductive dissociation reactions.^86^, ^87^, ^88^, ^89^ The mentioned reactions are highly chemoselective and have been demonstrated to proceed in complex environments such as cells.

**Figure 4:**
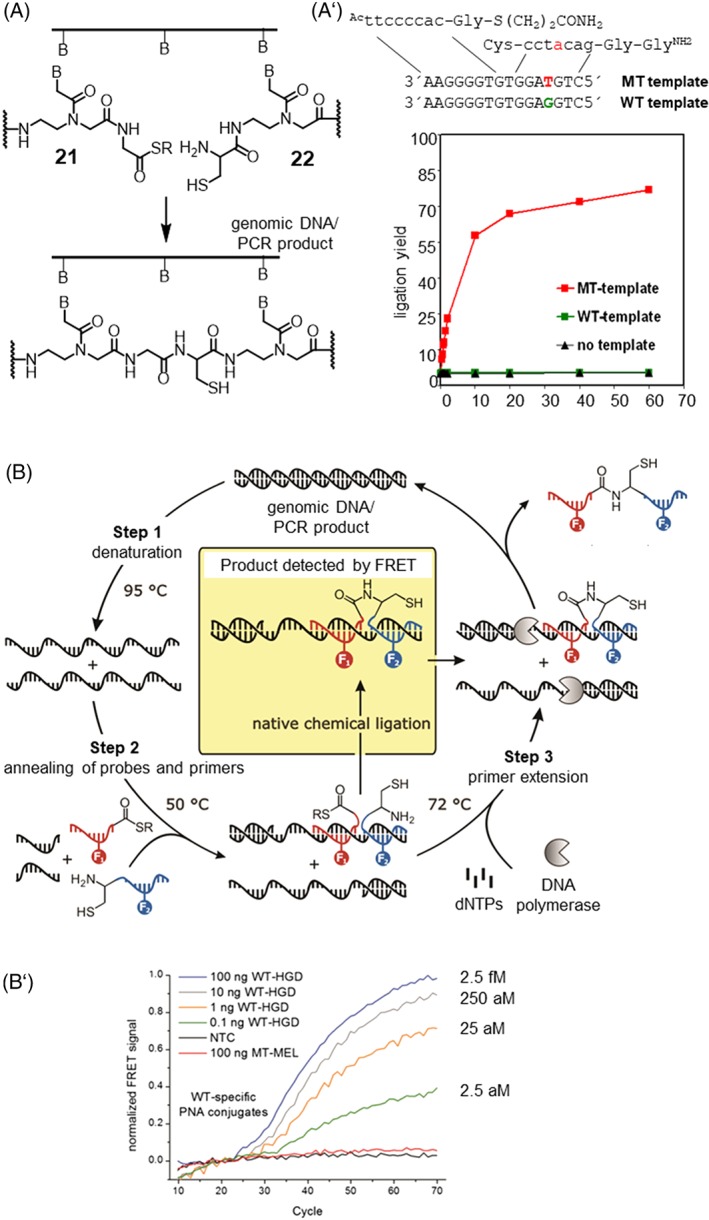
A, Principle of nucleic acid–templated native chemical ligation (NCL) of peptide nucleic acid (PNA). A′, Reaction time course of templated PNA NCL on matched and mismatched template (conditions: 1 μM PNA probes and templates, 100 mM Na_2_HPO4, sat BnSH, pH 7.4, 25°C). B, DNA template NCL during PCR (reproduced from Roloff and Seitz^90^ with permission from The Royal Society of Chemistry). B′, Amplification plots obtained by determining the normalized fluorescence resonance energy transfer Föster resonance energy transfer (FRET) signal upon ligation during polymerase chain reaction (PCR) in the presence of varying amounts of matched (Raf‐wt human genomic DNA, WT‐HGD) or mismatched (Raf‐T1799A‐mt human genomic DNA, MT‐HGD) DNA target (PCR conditions: 400 nM forward primer, 50 nM reverse primer, 200 mM dNTPs, 2.5 mM MgCl_2_, 300 nM PNA conjugates, 1 mM MESNA, 10 mM TRIS, pH 8.5 (at 25°C), 1 u Taq‐Pol; PCR protocol: 10 s at 95°C (step 1), 30 s at 50°C (step 2, detection), 20 s at 72°C (step 3))

Driven by the prospect of enabling a nucleic acid instructed peptide synthesis, we explored NCL reactions. In our first example of such a reaction, we equipped PNA molecules with a C‐terminal glycine thioester **21** or an N‐terminal cysteine residue **22** (Figure 4A).^91^, ^92^, ^93^ The nonionic DNA analogue PNA was chosen owing to its compatibility with peptide synthesis. Adjacent hybridization of two PNA strands with the nucleic acid template brings the reactive groups in close proximity and accelerated the NCL by 10^3^‐fold in initial reaction rates. The reaction showed a remarkable target specificity: A mismatch of a single nucleotide within the target strand nearly abolished the template effect, and the NCL ceased to proceed (Figure 4A′).^92^ To demonstrate the method's exquisite chemoselectivity, we interfaced the templated ligation with the polymerase chain reaction (PCR, Figure 4B).^90^ The PCR provides a formidable challenge to NCL chemistry. The thioester should remain intact despite the high temperatures and slightly basic pH applied in the PCR process. Nevertheless, the templated reaction must occur rapidly within the rather short time available when the target strand is accessible in the primer extension phase of PCR. We found that the use of β‐alanine (rather than glycine) thioesters greatly decreased the vulnerability against hydrolysis without detriment to the speed of templated ligation. The “in‐PCR set‐up” allowed DNA template synthesis starting from attomolar concentrations of target (Figure 4B′). We used the templated ligation to determine the number of triplet repeats in Huntington DNA, which at lengths >36 repeats cause chorea Huntington disease.^94^ Middel et al used photocleavable PNA templates to direct NCL to a glutamic acid side chain.^95^ Very recently, Sayers et al reported a templated ligation between PNA‐linked selenoesters and selenocysteine.^96^ The reaction is the fastest nucleic acid–templated reaction to date and has been used to detect miRNA within cell lysates by means of a paper strip assay.

The need for stoichiometric amounts of target strand is a key issue of templated ligation chemistries. In real‐world scenarios, nucleic acid molecules occur in rather low quantities. PCR is not an option for reactions that ought to proceed in biological samples. Ideally, the templated reaction should provide for turnover in the target strand.^97^, ^98^ In this case, each target molecule would instruct the formation of many product molecules. However, nucleic acid–templated ligation reactions suffer from product inhibition. The ligation products contain more nucleobases, and therefore, ligation products typically have higher affinity for the target strand than the reactive conjugates prior to reaction. One approach to reduce product inhibition in nucleic acid–programmed ligation reaction involves the integration of units that destabilize the product‐target complex. Careful optimization of the ligation site such as the replacement of a glycine‐cysteine by a glycine‐isocysteine junction allowed improvements of turnover numbers.^93^ In another report, we described bifunctional PNA conjugates, which reacted in a two‐step reaction.^99^ Templated NCL was followed by a cyclization reaction. The cyclic products had lower template affinity than the ligation products prior to cyclization. Under thermocycling conditions, the two‐step ligation‐cyclization reaction provided two to three times more product than the “ligation only” reaction. However, thermal cycling is, again, not an option for reactions designed to occur in experiments that include cellular material such as in lysates or fixed/live cells.

**Figure 5:**
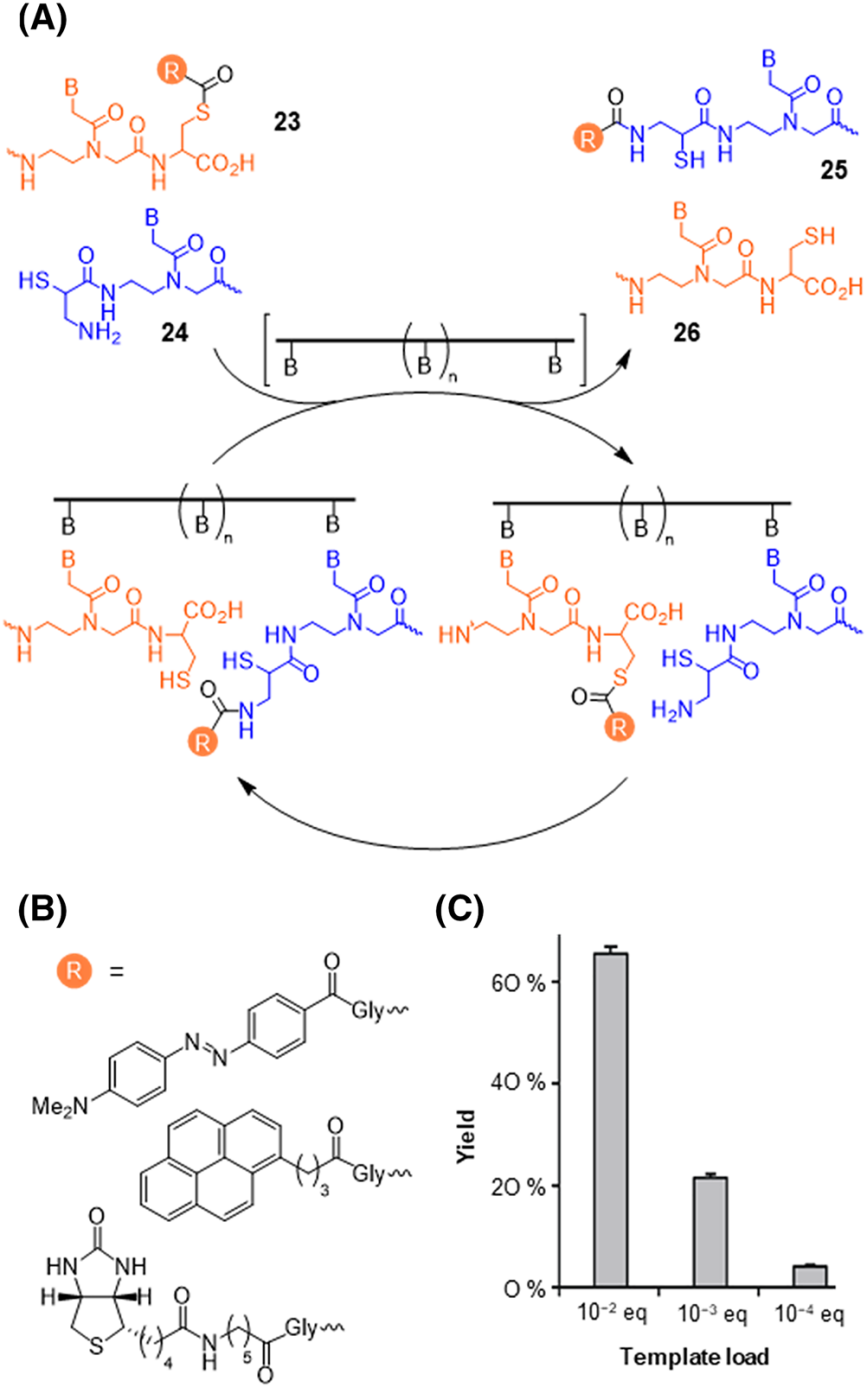
A, Nucleic acid–templated acyl transfer reaction. B, Reporter groups used in templated acyl transfer reactions. C, Yield of transfer reaction between FAM‐AEEA‐tcttccccac‐Cys (Gly‐Dabcyl) and iCys‐cctacag‐Lys (AEEA‐TAMRA) at substoichiometric amounts of template 5′‐GCCGCTGTAGGTGTGGGGAAGAGT‐3′ (conditions: 100 nM probes, 10 mM NaH_2_PO_4_, 200 mM NaCl, 1 mM triscarboxyethylphosphine (TCEP), 0.1 mg/mL Roche blocking agent, pH 7.0, 32°C)

To ease the problem of product inhibition, we conceived a templated NCL‐like reaction that avoids ligation of the two reactive PNA strands. Instead, the reaction involved the transfer of a thioester‐linked acyl unit from a donor conjugate to an acceptor conjugate (Figure 5).^100^ The reactions still proceed in analogy to the NCL reaction. However, the thiol component of the donor conjugate **23** not only serves as a leaving group but in addition also includes functionality, i.e., a DNA/RNA recognition unit. Because the products **25** and **26** of the acyl transfer reaction contain as many nucleobases as the starting materials, the reaction can proceed under conditions of dynamic strand exchange, which allows dissociation of product from the target strand and association of starting materials. As a result, the target strand may act as a catalyst. In the first example, the reaction was designed to trigger the transfer of a fluorescence quenching Dabcyl dye from a fluorescein‐labelled donor to a rhodamine‐labelled acceptor strand (Figure 5B).^100^ The concomitant restoration of fluorescein emission and decrease of rhodamine emission provided a facile read‐out in real time. At 1 nM target strand and 100 nM reactant probes, the reaction delivered 68 nM product (Figure 5C). Considering that 3.4 nM were obtained in the absence of target, this corresponds to 65 turnovers. Further decreases of the target concentration to 10 pM allowed the reaction to proceed with 400 turnovers.

In subsequent studies, we demonstrated the versatility of the acyl transfer reaction. We showed a DNA‐promoted transfer of pyrene and an RNA‐promoted transfer of biotin onto a His_6_‐tagged acceptor.^101^, ^102^ After immobilization onto Ni‐coated microtiter plates, transferred biotin was quantified by means of a horseradish peroxidase‐streptavidin. The setup allowed the detection of 500 attomol RNA target. Recently, we used the reaction in the RNA target–promoted transfer of fluorophores onto semiconductor quantum dots.^103^, ^104^ We showed that the acyl transfer reaction is not restricted to PNA‐based reaction systems but also proceeds with reactive DNA conjugates.^105^

**Figure 6:**
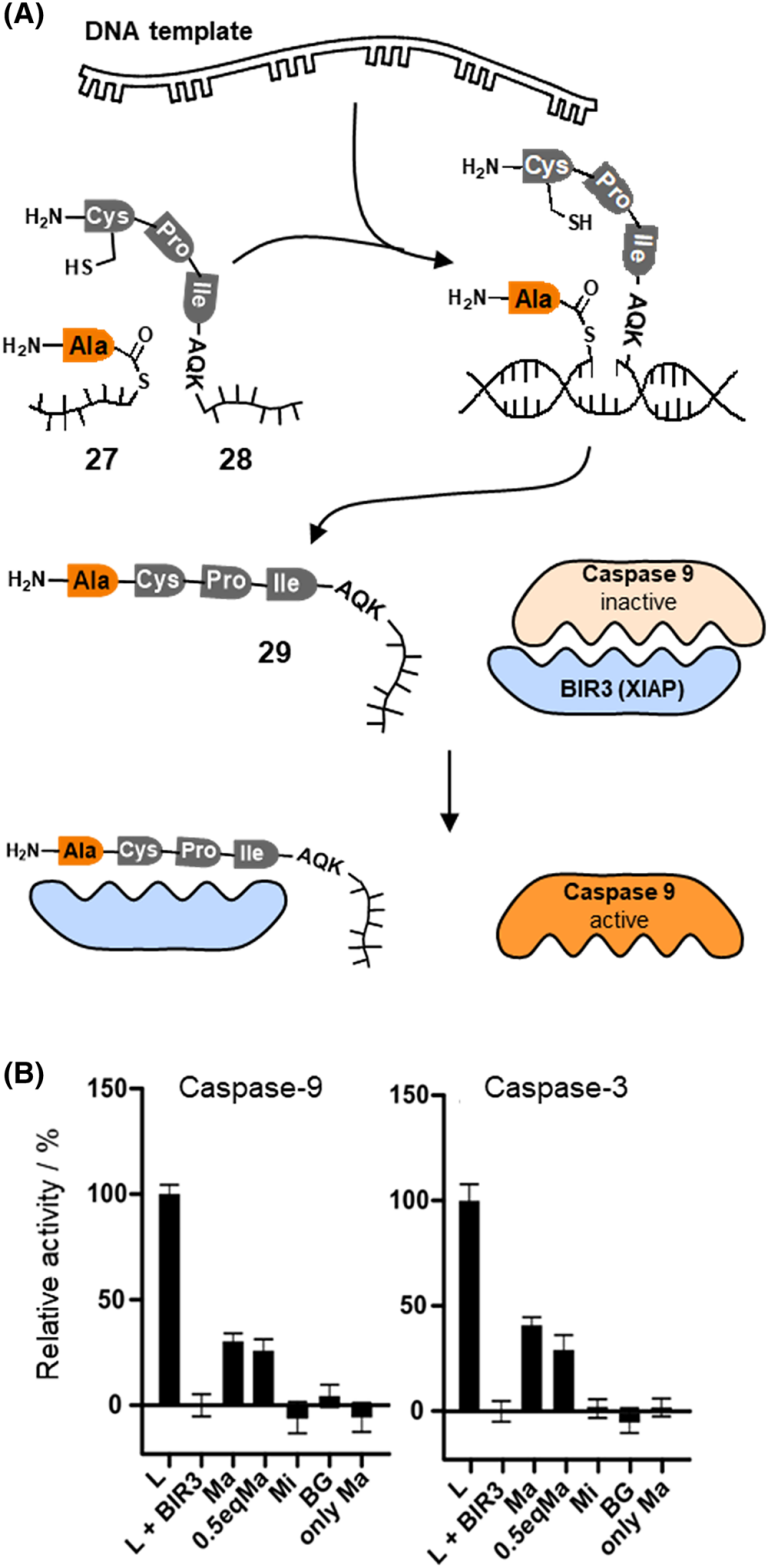
A, Templated alanyl transfer induces the formation of peptide **29**, which displaces caspase‐9 from the XIAP‐BIR3 domain. B, Relative activity of caspase‐9 and caspase‐3 in HEK293 cell lysate (L) and after addition of the XIAP‐BIR3 domain (L + BIR3). Addition of probes 27 and 28 in the presence of matched template (Ma) led to caspase reactivation. No reactivation was observed in the absence of template (BG), with mismatched template (Mi) or in template only experiments (only Ma) (reproduced from Erben et al^107^; copyright 2011 Wiley)

At the outset of this chapter, I described the idea of developing systems that read RNA and translate the recognition event into a reaction product that interferes with cellular processes. Most of the reactions pertinent to this idea belong to the category of cleavage reactions. Typically, bioactive molecules are released from inactive, prodrug‐like forms by some kind of dissociative chemistry such as hydrolysis, tetrazine‐mediated cleavage, reduction‐triggered fragmentation, or photoinduced cleavage.^16^ The aforementioned acyl transfer offers the prospect of building up drug‐like molecules by bond‐forming rather than bond‐cleaving reactions. The first example was described by Erben et al.^106^, ^107^ The idea was to translate nucleic acid information into the output of peptide molecules that interfere with disease‐related protein‐protein interactions. In this example, a DNA target strand triggered the transfer of an alanine residue from thioester‐linked PNA conjugate **27** onto tripeptides or hexapeptides (Figure 6A). Interestingly, the length of the acceptor peptide **28** had little effect on the transfer rate. More than 60% product was obtained in less than 30 minutes. The formed peptide was designed to disrupt the interaction between the caspase‐9 and the BIR3 domain of the X‐linked inhibitor of apoptosis protein XIAP. This interaction holds the caspase‐9 in an inactive state and prevents activation of apoptosis in cancer cells. Peptides containing an N‐terminal Ala‐Val‐Pro‐Ile tetrapeptide motif displace caspase‐9 from the XIAP‐BIR3 domain. We found that a Val → Cys change is tolerated. Accordingly, a DNA‐triggered synthesis of the Ala‐Cys‐Pro‐Ile in **29** allows the reactivation of the initiator caspase‐9 and, subsequently, of the executioner caspase‐3. The reaction was performed in total lysate of HEK293 cells, which contained the DNA target, if added, and BIR3 to emulate the action of XIAP in cancer cells. The addition of matched DNA target rescued the activity of caspase‐9 and caspase‐3 by 27% and 45%, respectively (Figure 6B). No restoration of caspase activity was observed when single base mismatched DNA target was added. This is testimony for the high target specificity of the templated acyl transfer.

**Figure 7:**
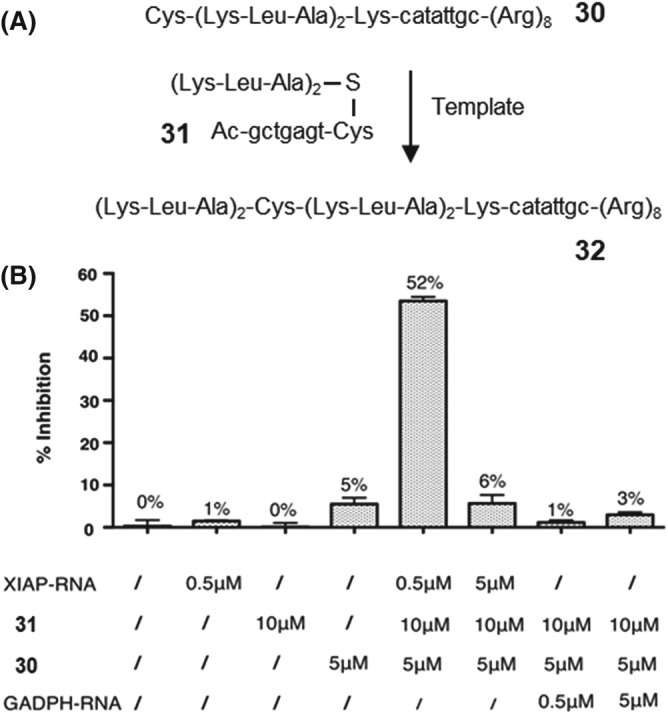
A, RNA‐templated peptidyl transfer reaction between **30** and **31** leads to conjugate **32**, which is cyctoxic when produced in excess to template. B, Inhibition of HeLa cell proliferation (MTS assay) after treatment with reactive probes in the presence of matched (XIAP‐RNA) and mismatched (GAPDH‐RNA) template (reproduced from Vazquez and Seitz^108^ with permission from The Royal Society of Chemistry)

To provide unambiguous proof for the catalytic activity of a nucleic acid target, we conceived a reaction system that requires turnover in an RNA target to evoke bioactivity. The reaction involved the transfer of a hexapeptide from **31** onto the octapeptide‐PNA‐octaarginine conjugate **30** (Figure 7A).^108^ Once delivered into cells, the KLAKLACKLAKLAK sequence exhibits cytotoxic properties, probably by disrupting the mitochondrial membrane. For this activity, the cytotoxic peptide needs the help of the cell penetrating octaarginine unit. However, the RNA target added to instruct product formation outside of cells masks the positive charges required for cell penetration and mitochondrial membrane disruption. As a result, HeLa cells survived the incubation with a product mixture formed on stoichiometric RNA target (Figure 7B). By contrast, greater than 50% of the cells died when the reaction was performed on 0.1 equivalents RNA target. Because of turnover in target, the cytotoxic peptide‐PNA‐R8 molecules **32** are formed in excess of the RNA target molecules. Without capture by the RNA target, the excess product is free to act on cells. Neither fragment showed cytoxicity at the concentrations applied. The reaction proceeded, again, in a target‐specific manner as observed by the lack of cell killing upon addition of target‐unrelated GAPDH‐RNA. This experiment provides biological proof that the concentration of molecules formed in a templated peptidyl transfer reaction can exceed the concentration of the template.

In a subsequent study, Di Pisa et al analyzed the RNA‐triggered peptidyl transfer reaction in greater detail.^109^ The authors evaluated the RNA‐programmed synthesis of a 16‐mer peptide that allows inhibition of the antiapoptotic Bcl‐x_L_ protein. It was found, perhaps surprisingly, that the length of the donor and acceptor peptides played minor roles. In stark contrast, the nature of the amino acid at the C‐terminal thioester was critical. Out of eight amino acids tested, glycine and alanine allowed for the highest target‐induced rate acceleration (4700‐ and 3000‐fold, respectively) whereas valine and isoleucine reacted reluctantly. The reaction rates under turnover conditions were influenced by the distance between the annealing sites (optimum with two unpaired spacer nucleotides) on the RNA target and the affinity of the PNA units for the target. The authors also assessed the affinity of transfer products for the Bcl‐x_L_ protein. This allowed the authors to identify the minimum concentration of RNA target required to inhibit Bcl‐x_L_ by >50%. They found that 10nM RNA target should be sufficient. Given the approximately 3000‐fL volume of a HeLa cell, this corresponds to 2000 copies. Some cancer genes are expressed at even higher copy numbers, which suggests that the templated chemistry should be efficient enough for a gene expression directed perturbation of living systems. A key requirement is, however, the availability of methods that allow the reliable cellular delivery of the conjugates.

## 6 TERMOLECULAR AND MULTIMOLECULAR ASSEMBLIES FOR NUCLEIC ACID–PROGRAMMED SPATIAL SCREENING OF RECEPTOR TARGETS

Many receptor systems offer more than one binding pocket for enhanced interactions with multivalent ligand systems. For example, lectins frequently form multimeric superstructures for interactions with multiply presented carbohydrate ligands on cell surfaces.^110^, ^111^ Important signal transducing receptors such as receptor tyrosine kinases form dimers.^112^ In many cases, multimeric receptor clusters are discussed. Of note, interactions between two cells or between a cell and bacteria or viruses are multivalent by nature, owing to the involvement of many receptor and ligand molecules on each side. The simultaneous engagement of multiple receptor‐ligand pairs strengthens the interactions, which can then occur at concentrations below the dissociation constant of the monovalent receptor‐ligand complex. Multivalency‐enhanced binding is not limited to recognition events on the cell surface. Many intracellular proteins contain more than a single protein‐protein interaction domain. For example, some kinases and phosphatases arrange Src homology‐2 (SH2) domains in tandem to allow tight interactions with proteins that contain two phosphotyrosine consensus motifs.^113^ Proteins involved in RNA splicing such as FBP21 comprise two WW domains for recognition of proline‐rich peptides,^114^ and epigenetic readers are often arranged in tandem.^115^

**Figure 8:**
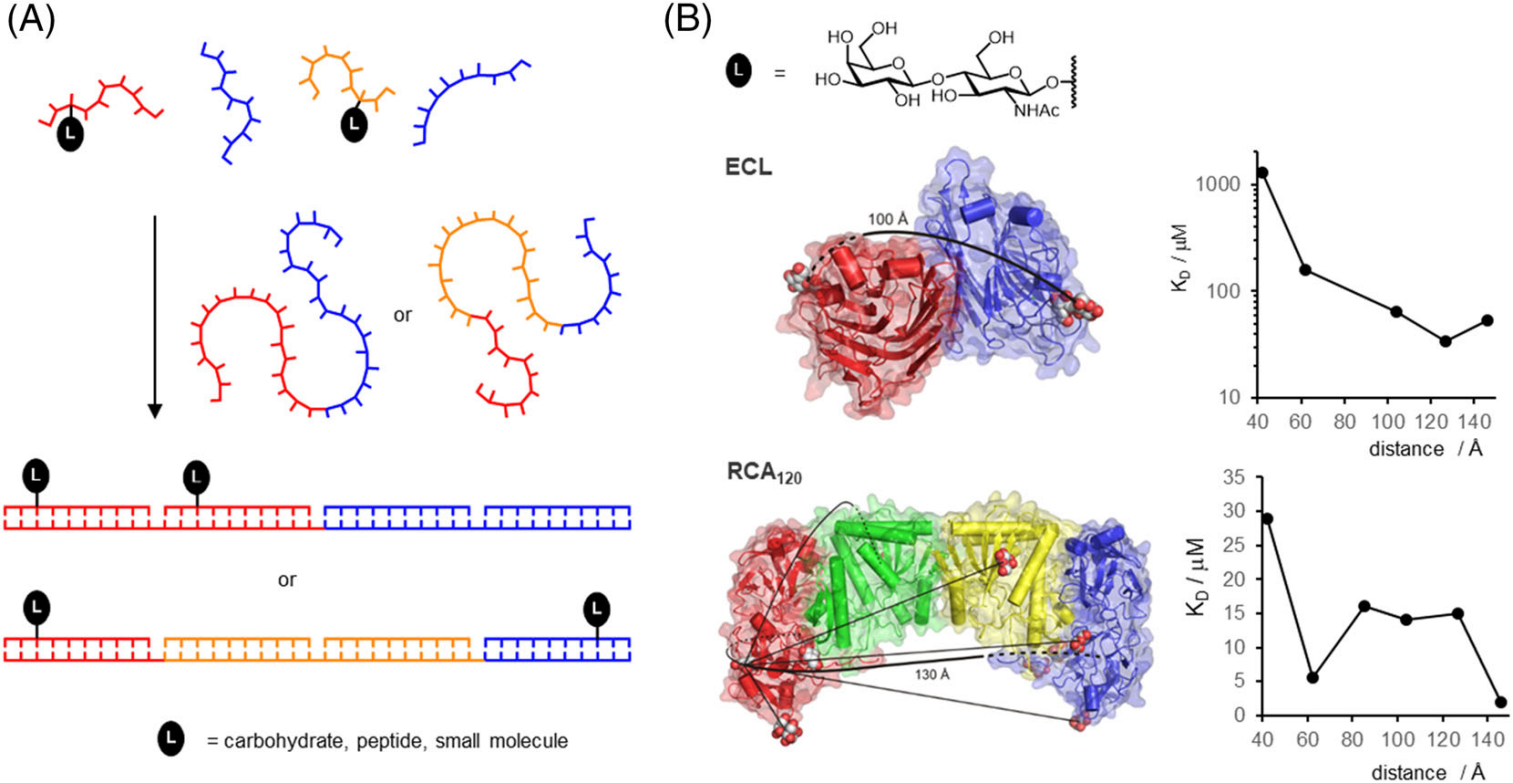
A, DNA‐templated self‐assembly with nucleic acid–ligand conjugates provides control over the valency and spatial arrangement of ligand displays. B, DNA‐programmed spatial screening of interactions between bivalent displays of LacNAc and Erythrina cristagalli lectin (ECL, top) or Ricinus communis agglutinin (RCA_120_, bottom) reveals distinct distance‐affinity relationships

The extent of the binding enhancement provided by multivalency depends, among other factors (*vide infra*), on the number and orientation of ligands on the multivalent display that should match the arrangement of binding pockets offered by the receptor system.^32^, ^116^ Often, the structure of the receptor system is unknown, and as a result, it is unclear how a multivalent ligand display should be designed in order to allow for tight interactions at acceptable ligand economy. We reasoned that DNA would be an ideal scaffold for controlling the number and orientation of ligands because (a) hybridization of complementary strands provides full control over the valency of the ligand display and (b) sequence‐programmed self‐assembly of duplex and higher order structures is well established allowing Ångstrom‐precise positioning of ligands. In an approach, which we termed DNA‐programmed spatial screening, the distance between the ligands is systematically varied (Figure 8A).^117^ Assemblies that arrange the ligands in an orientation that matches the arrangement of binding sites will provide the highest affinity for the receptor system under scrutiny. Therefore, spatial screening provides structural information about the receptor system.

Pioneering studies from Baird involved the DNA‐programmed bivalent presentation of haptens to characterize bivalent binding by antibodies.^118^ Matsuura et al examined the interactions between high molecular weight DNA‐galactose cluster on lectin recognition.^41^ Gorska et al described the DNA‐programmed presentation of bivalent and monovalent dimannosides and trimannosides.^119^ By examining binding to the antibody 2G12, which broadly neutralizes human immunodeficiency virus (HIV), the authors concluded that DNA‐guided presentation of carbohydrates emulates the complex carbohydrate epitopes found on gp120 of HIV. We conjugated a single *N*‐acetyllactosamine (LacNAc) unit with PNA and used hybridization with DNA to align one to four PNA strands.^117^ Each nick site between the annealed PNA strands can be regarded as a hinge that enables torsions around the helical axis and facilitates bending. The torsional flexibility is important to avoid a scenario where the distance between the two ligands would be optimal for bivalent interaction with the receptor but access would be blocked owing to presentation on opposite sides of the helix. We used the sequence‐programmed assembly to position the glycan residues in distances between 42 and 146 Å. The highest affinity for the *Erythrina cristagalli* lectin (ECL) was obtained when the glycan residues were displayed by using a 104‐ to 127‐Å long connector region (Figure 8B, top).^117^ ECL orients the sugar binding pockets on opposite sides in 65 Å Euclidian distance. We inferred that the 104‐ to 127‐Å long scaffolds are required to allow bending over the convex surface of the bean‐shaped protein. In subsequent studies, we analyzed bivalent interactions with the tetrameric Ricinus communis agglutinin (RCA_120_).^120^ According to crystal structure analysis, the RCA_120_ tetramer arranges the sugar binding sites in 120 Å Euclidian distance (Figure 8B, bottom). The DNA‐programmed spatial screen revealed that the highest (250‐fold) enhancement of binding affinity was observed when the two LacNAc residues were separated by 146 Å, which, again, matches the distance required to adapt to the convex surface.

**Figure 9:**
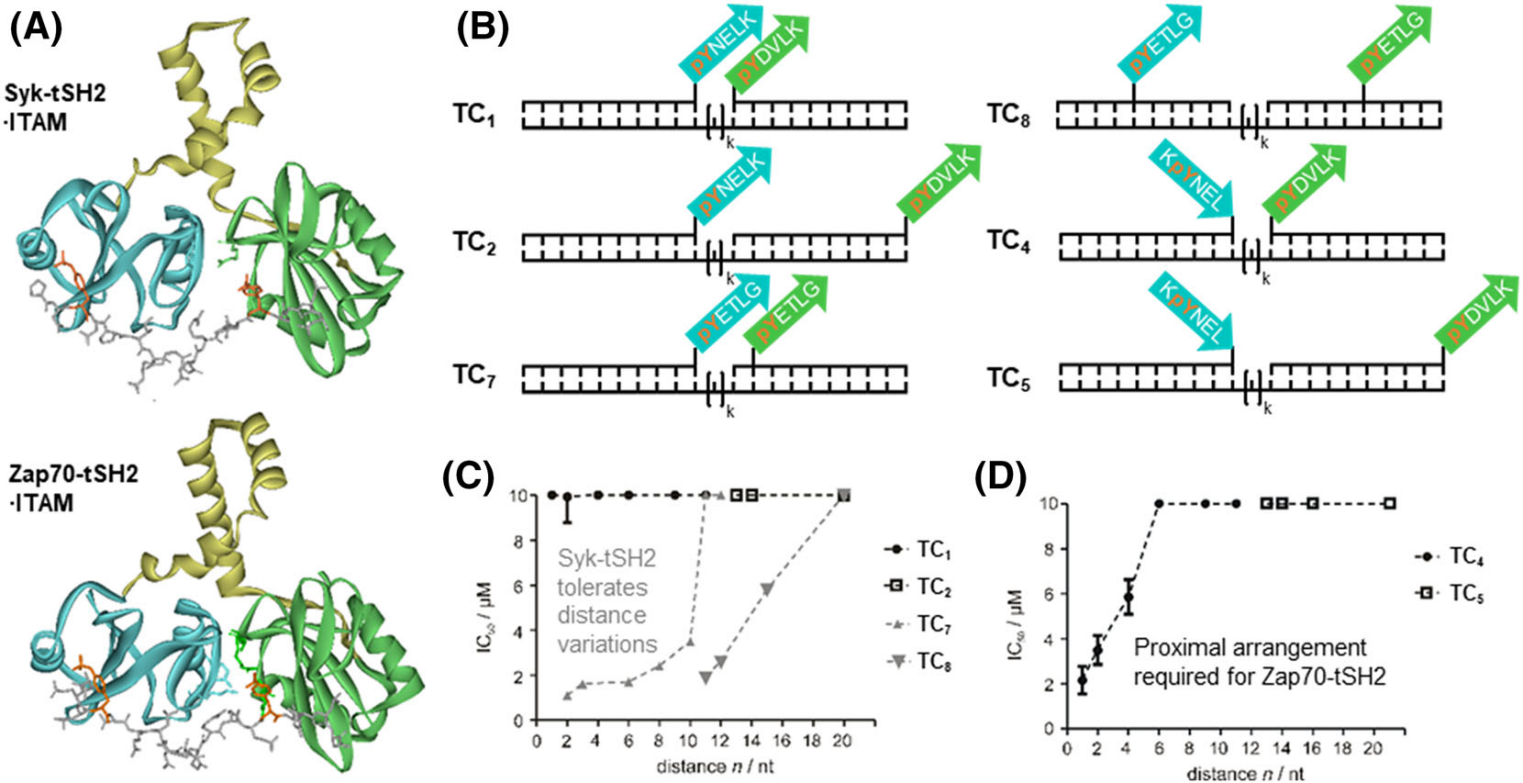
A, Crystal structures of Syk and Zap‐70 tSH2 domains in complex with double phosphorylated immunoreceptor tyrosine–based interaction motifs (ITAMs) from the CD3ε chain of the B‐cell receptor complex or the ζ‐chain of the T‐cell receptor, respectively (from PDB ID 1A81 and 2OQ1, reproduced from Marczynke et al^121^; copyright 2017 American Chemical Society). B, DNA‐peptide complexes used for DNA‐programmed spatial screening. Distance‐affinity relationships for interactions with Syk tSH2 (gray) or Zap‐70 tSH2 (black) obtained by spatial screening with complexes displaying peptides in C, same strand orientation or D, opposing strand orientation

The DNA‐based spatial screening was used to identify selective binders of two closely related members of the Src family of tyrosine kinases.^121^ The spleen tyrosine kinase (Syk) and the ζ‐chain associated protein kinase (ZAP‐70) bind with nanomolar affinity to the immunoreceptor tyrosine–based interaction motifs (ITAMs) of engaged B‐cell and T‐cell receptor complexes, respectively.^122^ The ITAMs contain the diphosphorylated consensus sequence pYXXI/L(X)_6‐8_pYXXI/L. For the firm interaction and full kinase activation to occur, Syk and ZAP‐70 arrange two Src homology 2 (SH2) domains in tandem (Figure 9A). Syk and ZAP‐70 play crucial roles in the activation of B and T cells, respectively. Binding experiments show that the tandem SH2 (tSH2) domains cannot distinguish between ITAM peptides from B‐cell and T‐cell receptor complexes. This poses a specificity challenge for the design of subtype‐specific binders and raises the question how differential activation can be achieved when both kinases are coexpressed. To address this question by DNA‐based spatial screening, the pYXXL motifs were conjugated with oligonucleotides and assembled to form bipartite ITAMs (Figure 9B).^121^ Syk tSH2 showed a broad substrate scope and interacted firmly with a number of different assemblies (Figure 9C, gray curve). This indicated a remarkable flexibility of the Syk tSH2 interdomain that accepts varied orientations of the individual pYXXI motifs until a critical threshold length of approximately 50 Å was reached. In stark contrast are the binding properties of the ZAP‐70 tSH2 domain (black curve, Figure 9C and 9D), which requires a proximal arrangement of the pYXXI motifs in defined orientation of the phosphopeptide strands. The DNA‐spatial screen on one hand exposed the different binding mechanism of seemingly similar protein‐protein interaction domains. On the other hand, the investigation also showed how constraining of ITAM motifs by secondary structure could contribute to differential activation of the kinases. Furthermore, the results guided the design of unimolecular phosphopeptide conjugates with submicromolar affinity, which discriminate between ZAP‐70 and Syk by one order of magnitude in affinity.

**Figure 10:**
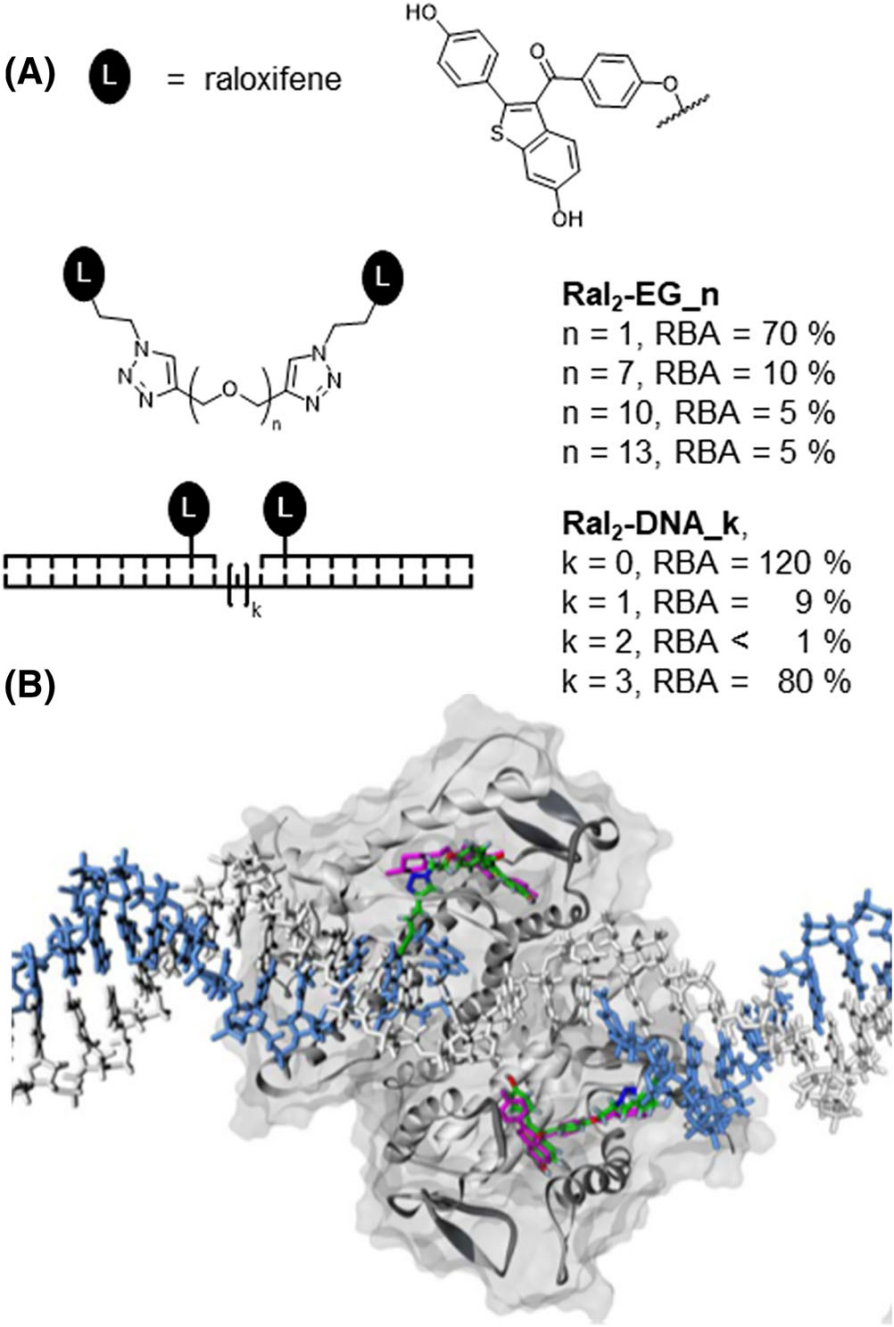
A, Relative binding affinity (RBA, relative to estradiol) of bivalent raloxifene displays for the estrogen receptor α. Nonconjugated raloxifene has RBA = 30%. B, Docking (reproduced with permission from Abendroth et al^124^; copyright 2011 Wiley) of **Ral**_**2**_ **‐DNA_3** (raloxifene shown in green) to the ERα ligand binding domain with the depiction of unconjugated raloxifene (magenta) in a cocrystal structure (PDB ID: 2R6W)

An often less noticed asset of DNA templates is their high solubility. This property is particularly advantageous for the multivalent presentation of hydrophobic ligands. In one case study, we and collaborators analyzed the interactions of the estrogen receptor (ER) and bivalent estrogen analogues.^123^, ^124^ The ER is a key regulator of gene expression and a frequently addressed drug target. Binding of ligands to the ER ligand binding domain (LBD) stabilizes the dimeric state, but owing to a subnanomolar dimerization constant, the ER dimer exists also in the absence of ligand.^125^ This finding has sparked the development of bivalent ER ligands. However, the design of high affinity bivalent ER ligands has proven challenging, and most of the bivalent ligand systems reported have lower ER affinity than the monovalent ligands.^123^, ^126^, ^127^ Typically, the hydrophobic ER ligands were linked via flexible tethers such as oligoethyleneglycol or oligomethylene spacers. For example, raloxifene binds the human ERα with 30% relative binding affinity (RBA) on the basis of the affinity provided by estradiol, whereas binding of bivalent systems **Ral**_**2**_ **‐EG_n** (*n* = 7, 10, 13) is characterized by RBA = 5%‐10% (Figure 10A). NMR and UV spectroscopic analyses hint at hydrophobic interactions between the ER ligands that may penalize binding to the ER. Furthermore, tethers such as oligoethyleneglycol can fold back and wrap around the ER ligand.^123^ Such interactions should not occur with DNA‐based spacers. Indeed, termolecular DNA assemblies, in which two appended raloxifene units were positioned in three or six nucleotides distance (**Ral**_**2**_ **‐DNA_k**, *k* = 0, 3), bind the ERα with 120% and 80% RBA, respectively.^124^ Molecular modeling suggested that the six‐nucleotide‐spaced display can bridge the 35 Å distance between the canonical estrogen binding sites (Figure 10B). However, the three‐nucleotide spacer is too short to span this distance. Crystal structure analysis and docking studies suggested the presence of secondary binding sites 17 Å away from the canonical binding site. We speculated that the three‐nucleotide‐spaced arrangement picks up interactions with this hydrophobic patch. On the basis of this assumption, we tethered two raloxifene units in **Ral**_**2**_ **‐EG_1** via a spacer too short for bridging the canonical binding sites but of sufficient length to allow engagement of one canonical and one secondary binding site. This approach resulted in increased ER affinity (RBA = 70%). Spectroscopic measurements suggested that the short tether does not permit homophilic raloxifene interactions. The knowledge obtained in these studies was used for the design of high affinity fluorescent ERα binders.^128^

**Figure 11:**
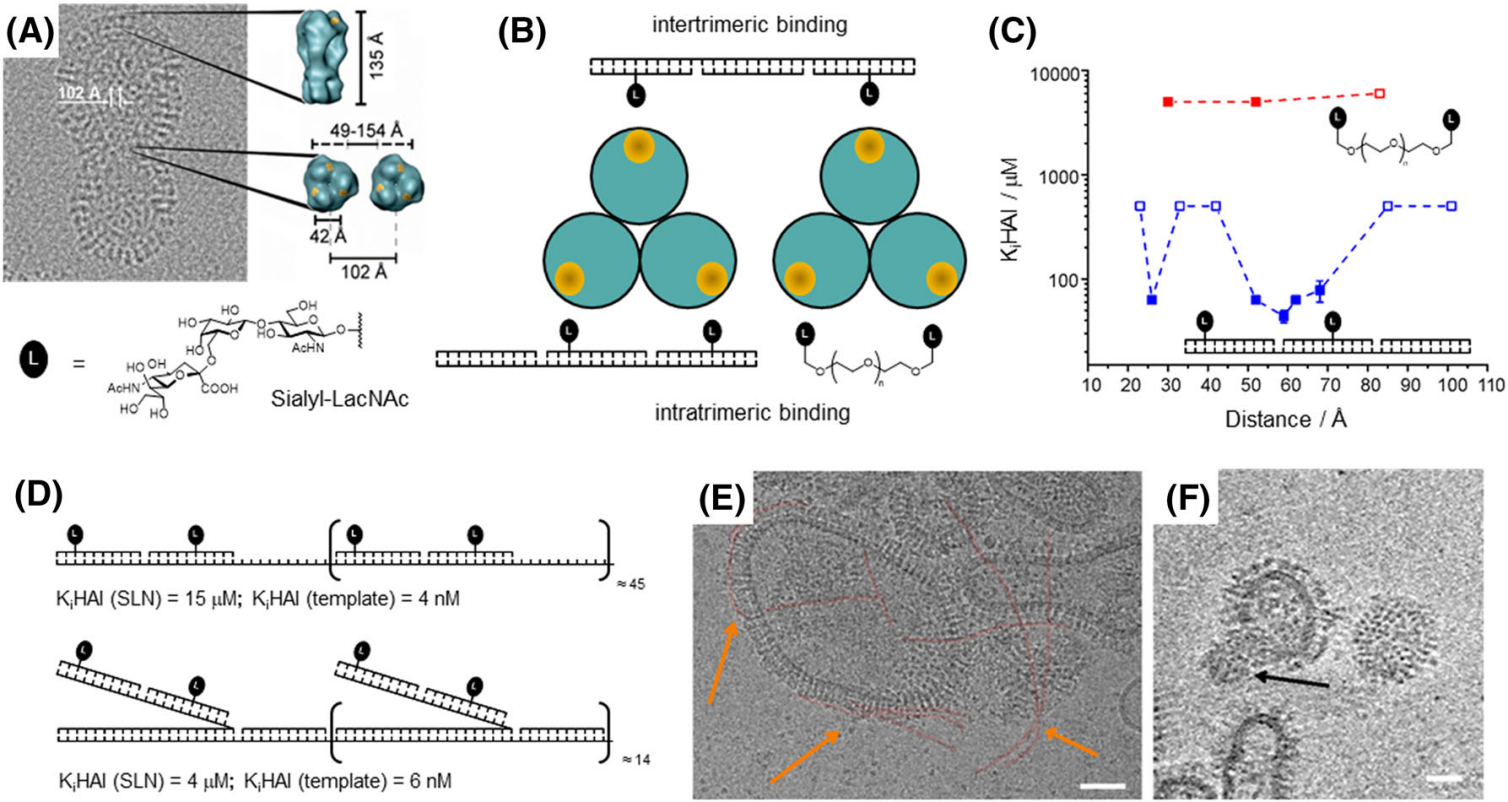
A, Cryo‐electron micrograph of human influenza virus (X31) and depiction of trimeric hemagglutinin (HA) with position of canonical sugar binding sites marked in yellow. The distance between two binding sites within a single HA trimer was extracted from crystal structure analysis (PDB ID: 1HGG, reproduced from Bandlow et al^129^; copyright 2017 American Chemical Society). The distances between two adjacent HA trimers were determined by cryo–transmission electron microscopy (cryo‐TEM) analysis. Given free rotation of HA trimers, two canonical glyco binding sites on adjacent HA trimers could be arranged in 49 to 154 Å distance. B, Bivalent displays for probing of intertrimeric and intratrimeric HA interactions. C, Distance‐dependent inhibition of influenza X31 hemagglutination by bivalent Sialyl‐LacNAc conjugates. Open squares show data for the highest concentration of conjugate applied at which hemagglutination was still not inhibited (reproduced from Bandlow et al^129^; copyright 2017 American Chemical Society). D, Concatenation of optimized bivalent Sialyl‐LacNAc displays by linear (top) or branched (bottom) hybridization with long DNA templates produced by rolling circle amplification. The values K_i_HAI are based on the concentration of sugar (SLN) or template required to fully inhibit hemagglutination. E, Cryo‐electron micrograph of DNA‐concatenated SLN incubated with IAV X31 for 30 min and embedded in vitreous ice. Linear spaghetti‐type structures are highlighted in red and orange arrows. F, Slice of the reconstructed 3D volume of tilt series showing a virus‐bound wool‐type SLN concatemer. Scale bars: 50 nm. E and F reproduced from Bandlow et al^130^; copyright 2019 Wiley

**Figure 12:**
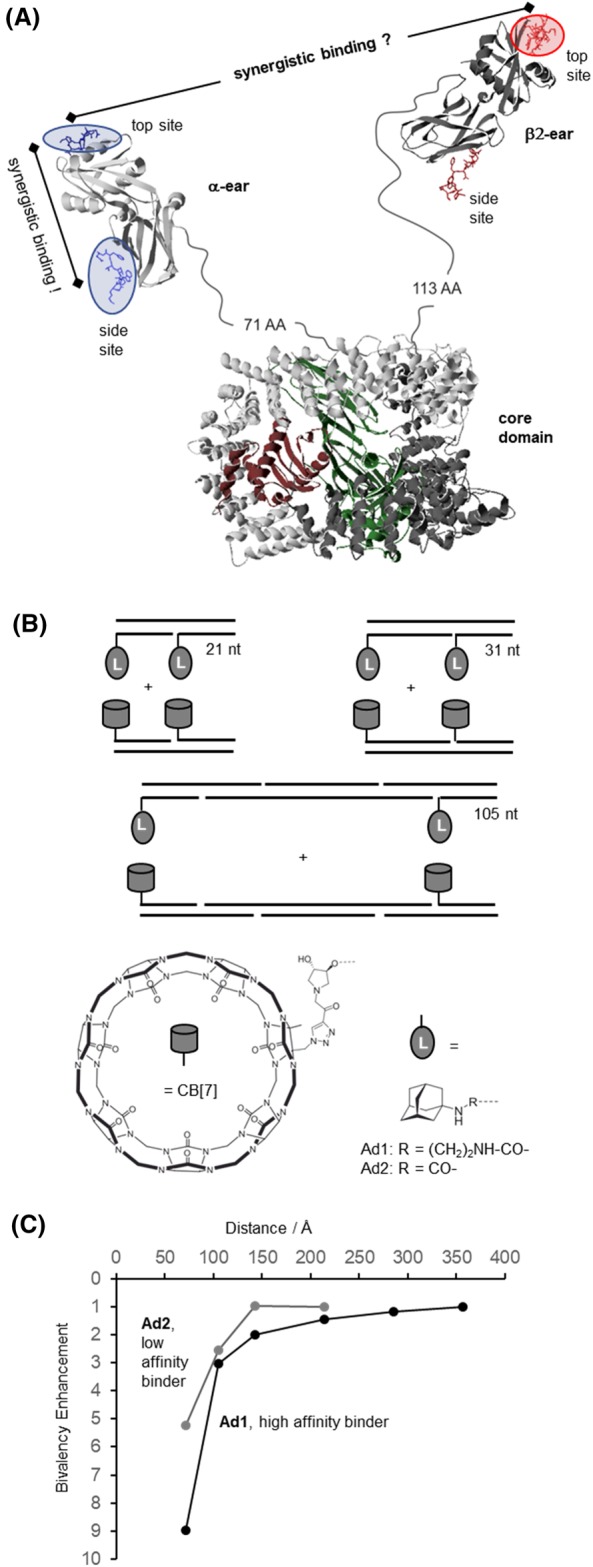
A, Graphical presentation of the heterotetrameric adaptor protein AP‐2 in complex with peptides that bind to the top and side sites of the ear domains (model based on PDB IDs 2VGL, 2VJO, 2G30, and 3HS9, modified from Dubel et al^137^; copyright 2019 Wiley). B, Representative examples of cucurbituril CB^7^– and adamantane‐DNA conjugates used to probe the distance‐stability relationship of complexes formed upon bivalent interaction. C, Distance dependency of bivalency‐enhanced interactions between distance‐matched CB^7^ and adamantane displays (bivalency enhancement = K_d_ (mono)/2·K_d_ (biv))

Viruses take advantage of multivalency‐enhanced interactions between protein‐based receptors and sugars in order to facilitate adherence to host cells. A well‐studied example is the influenza A virus (IAV) that offers hundreds of hemagglutinin (HA) trimers for enhanced recognition of cells displaying multiple sialylated galactose sugar units (Figure 11A). Driven by the medical need to prevent pandemic influenza infections, a variety of multivalent HA binders have been developed. A typical approach relies on multivalent presentation of glyco ligands from polymers,^131^, ^132^ dendrimers,^45^, ^133^ or nanoparticles.^134^, ^135^ It remains unknown which and how many of the several glyco ligands engage on binding and, therefore, these scaffolds do not provide information about the optimal spacing of HA ligands. In order to identify the criteria for enhanced binding at high ligand economy and learn about the arrangement of binding sites on the IAV particle, we used DNA‐programmed bivalent screening.^129^ We figured that a range of far reaching scaffolds would be required to assess the potential for enhanced interactions upon bridging of two sugar binding sites within an HA trimer and across HA trimers on the IAV surface (Figure 11B). The study also included a comparison between distance‐affinity relationships provided by two kind of scaffolds, i.e., rigid, sequence‐programmable DNA‐type architectures and flexible polyethylene glycol (PEG), which presented the sialyl‐LacNAc ligands in 23 to 101 Å averaged distance. The combination of distance‐affinity measurements by microscale thermophoresis and hemagglutination inhibition assays with a theoretical analysis by statistical mechanics models revealed that PEG‐based scaffolds fail to provide affinity enhancements (Figure 11C, red curve). PEG is too flexible to raise the effective molarity of the glyco ligands to the millimolar concentrations required to enable the interaction with the 42 Å‐spaced, low affinity binding site (*K*_mono_ = 3 mM). The situation is entirely different with the DNA‐based scaffolds. The DNA‐based spatial screening exposed a bimodal distance‐affinity relationship for both soluble HA and HA on the IAV surface (Figure 11C, blue curve). One of the binding optima, indicated by 10^3^‐fold enhanced binding, was obtained when the two ligands were separated by 52 to 59 Å. This probably is the spacer length required to bend over the slightly convex protein surface. A second binding optimum was observed for complexes that presented the glyco ligands at a 26 Å distance. This pointed to a secondary binding site, which corroborated previous results from crystal structure analysis.^136^ The spatial screen also revealed a preference for bivalent recognition of sugar binding sites within a HA trimer rather than binding across HA trimers on the IAV surface. This conclusion was drawn from a comparison of experimentally observed binding data and modeling data. The latter would have suggested an affinity enhancement for complexes displaying glyco ligands in >80 Å distance, which was not observed experimentally. In a separate study, we explored the reach of bivalency‐enhanced binding (*vide infra*, Figure 12).^137^ We found that the reach is critically controlled by the strength of the monovalent interaction. This suggests that the millimolar *K*_d_ provided by the interaction between a single sialyl‐LacNAc and a single HA binding site is not sufficient to enable bridging across two HA trimers.

Recently, we extended the DNA scaffolds to allow the oligomerization of distance‐optimized binders with micromolar affinity.^130^ For this purpose, we assembled DNA sequence repeat motifs by means of rolling circle amplification (RCA). In this method, a DNA polymerase extends primers according to the information on a circular DNA template. RCA of 39 or 50 nt long circular DNA provided single strands containing ≈46 or ≈15 repeats, respectively. These strands were used for the concatenation of sialyl‐LacNAc conjugates (Figure 11D). The addition of the DNA template enabled a 10^4^‐fold reduction of the concentration of trisaccharide ligand required for full inhibition of IAV. The most effective complexes fully inhibited IAV at 10^−9^ M DNA template. Cryo–transmission electron microscopy (cryo‐TEM) showed that the assemblies form linear spaghetti‐type (Figure 11E) and cotton ball–like (Figure 11F) structures that are able to connect several adjacent HA trimers on the IAV surface.

Many biological recognition systems involve binding sites that are separated by more than 100 Å distance. Such a scenario should be typical for weakly expressed receptors on the cell surface. In addition, a multitude of adaptor proteins offers two or more binding sites for interaction with potentially multivalent ligand motifs. Two binding modes are possible: (1) multivalency facilitates the formation of a bimolecular complex or (2) multimolecular interactions induce cross‐linking. Binding mode 1 allows complex formation at concentrations below the *K*_d_ of the monovalent interaction. Binding mode 2 is the hallmark of adaptor proteins serving as landing hubs to recruit many proteins to one site. It is plausible to assume that a given biological recognition system is evolutionary optimized for one of the two options. However, what are the design criteria? This question emerged during studies of the adaptor protein AP‐2 (Figure 12A).^138^ The heterotetrameric adaptor complex 2 (AP‐2) recruits clathrin to membrane regions destined for clathrin‐mediated endocytosis.^139^ AP‐2 harbors two ear (also termed appendage) domains: the α‐ear and the β2‐ear. Each ear domain offers two binding groves, the top site and the side site, for interactions with peptide motifs in other endocytic accessory proteins.^140^ According to crystal structure analysis, the two sites are separated by <60 Å distance.^141^, ^142^ A DNA‐programmed spatial screen revealed that the two binding sites can cooperate and provide enhanced interactions with bivalent peptide displays.^138^ The situation was less clear for interactions involving the α‐ and β2‐ear domains. The distance between the two different domains is ill‐defined, because the ears are connected to the AP‐2 core via structurally disordered, 71 and 113 amino acid long hinge regions. At a distance this large, it was unclear whether both ear domains can synergize upon simultaneous interactions with a single binding partner. We used self‐assembled peptide‐DNA complexes simultaneously targeting the α‐ and β2‐ear domains to experimentally probe the requirements for heterobivalency‐enhanced AP‐2 binding. However, bivalent complexes presenting the binder peptides in 10 to 140 nt distance (≈34‐476 Å based on B‐type duplex geometry) failed to bind AP‐2 with higher affinity than monovalent controls.^137^ We concluded that the distance between the α‐ and β2‐ear domains is too large to allow bivalency‐enhanced interactions. To test this assumption, we designed a model system, which would provide full control over both, the receptor and the ligand system.^137^ For this purpose, we attached two cucurbit^7^uril CB^7^ units to one DNA scaffold complex and two adamantane ligands to another DNA scaffold (Figure 12B). Adamantane and CB^7^ form host‐guest complexes at nanomolar concentrations. The study included two distinct adamantane guests that differed by the CB^7^ affinity. By systematically varying the distance between the adamantane and CB^7^ on DNA scaffolds, we explored the distance reach of bivalency‐enhanced interactions between bivalent guests and bivalent hosts (Figure 12C). The study revealed that the affinity gain provided by bivalency is critically controlled by the strength of the monovalent interaction. The higher the stability of the host‐guest (or in other words, receptor‐ligand) interaction the longer the linker can be. Importantly, the reach of the bivalency enhancement is reduced with increasing flexibility of both the receptor and the ligand scaffolds. The fact that the scope of bivalency is controlled by the distance between the receptor‐ligand pairs and the strength of the monovalent receptor‐ligand interaction has important consequences for cross‐linking. We designed fluorescence‐labelled complexes that show fluorescence resonance energy transfer (FRET) upon multimolecular interactions (cross‐linking) between CB^7^ and adamantane displays. Fluorescence measurements revealed that the degree of cross‐linking inversely correlates with the strength of the monovalent interaction and the distance. In other words, the weaker the affinity of a receptor for the ligand and the longer the distance between the receptors and ligands, the more likely cross‐linking is to occur. Receptor systems destined to act as cross‐linkers should therefore arrange low affinity binding sites via long flexible tethers. This is the design feature of AP‐2. Conversely, for allowing formation of receptor‐ligand complexes at concentration below the monovalent *K*_d_, high affinity binding sites shall be connected via short and less flexible tethers.

## 7 CONCLUSION

Templated chemistry once was a domain of supramolecular and materials chemistry where the prime interest of research pertained to the template structure itself. The view taken in this review is different. The templating approach is seen as an enabling tool to help address and solve problems in bioorganic synthesis and chemical biology. Which problems? The key challenge for molecules used in chemical biology and medicinal chemistry is specificity. For example, a multitude of different lectins has weak affinity for very similar glyco ligands. Because of the oligomeric arrangements and cell surface expression of lectins, their interactions are multivalent by nature. However, attempts to increase the potency of inhibitors by simply maximizing the number of interacting ligands on a structurally ill‐defined scaffold are likely to fail. Such “shotgun inhibitors” will not be specific. Rather, the multivalent display should orient the ligands in arrangements that match the orientation of binding sites on the targeted lectin. This calls for high precision templating of ligands that is feasible by using scaffolds on the basis of structured protein and nucleic acid molecules. By coincidence, two recent developments encourage the exploration of such complex biohybrid materials. First, given the impressive repertoire of bioconjugation methods available to date, connections between small molecules and large biomolecules are no longer viewed as an unsurmountable obstacle. Second, in the era of biologicals, biomacromolecules are no longer the exception but rather a well‐accepted treatment modality. So why not give it a try and explore medicine on the basis of protein‐ and nucleic acid–based scaffolding of small molecule ligands? Each nucleic acid–ligand conjugate could remain comparatively small, but self‐assembly (perhaps on the target site?) could lead to structures that have the degree of stiffness to allow precision scaffolding. The scope of template‐induced improvements of affinity and specificity extends beyond multivalency of a single target. It does not require much imagination to apply the concept to the simultaneous targeting of different cell surface proteins. Given that the number of different cell surface proteins is a proxy for the uniqueness of a cell, this approach will increase the cell‐type specificity, a desideratum in molecular cancer therapy.

Another actively pursued approach pertains to the development of methods that hijack natively expressed biomolecules and repurpose their function to serve as templates that instruct the formation drug‐like molecules. In this approach, a drug would be formed in situ only in those cells that express the instructing templates. At the first glance, this sounds like science fiction. But an impressive report from the Winssinger group has shown that cell endogenous RNA molecules can be used to drive a fluorogenic reaction inside whole organisms. Now, the art is to conceive a templated chemistry that induces the formation of a highly potent drug‐like molecule. We, and others, are working on it.

Specificity also is a key issue in peptide synthesis and live cell protein labelling. It is, for example, desirable to be able to append any kind of fluorescence label within minimal time. An ideal method would allow multiplexing in order to follow the localization of and interaction between two or more proteins in real time. Labelling would ideally extend beyond the appendage of fluorophores. Imagine the opportunities if the labelling reaction was reversible or created a handle that provides control over the localization of a protein and its interaction with other proteins or ligands. Again, templates will be of help. Short peptides and small molecule scaffolds provide unique microenvironments, which allow chemical reactions to proceed with exquisite site specificity. For example, end‐of‐helix arrangements at coiled coil peptides provide high effective molarities and enable labelling reactions within seconds to minutes at low concentration of labelling agent. Currently, we are exploring orthogonal coiled coils and the use of PNA‐based tags as generically addressable landing platforms.

I have sketched research problems that drive the research in my lab. In hindsight, so many things seem to follow a logical plan. However, as much as I would like to be able to follow a strict plan, I have to admit that serendipity has its place and very often it is chemistry and biology itself which drags us towards certain research topics. This has happened, for example, with our unexpected excursion to radical chemistry in ligation auxiliaries. Our model studies on the limits of bivalency were the result of fruitless and frustrating attempts with biological material. I am grateful to the many individuals who made this never‐ending journey to specificity possible and to the award of the Max Bergmann Medal that recognizes their work.
